# Target product profile: diagnostic test for *Trypanosoma brucei gambiense*

**DOI:** 10.2471/BLT.23.290172

**Published:** 2023-06-15

**Authors:** Gerardo Priotto, Jose R Franco, Veerle Lejon, Philippe Büscher, Enock Matovu, Joseph Ndung'u, Sylvain Biéler, Dieudonné Mumba, Nick Van Reet, Paul Verlé, Vincent Jamonneau, Pere P Simarro, Augustin Kadima Ebeja, Dieudonné Sankara, Daniel Argaw Dagne

**Affiliations:** a Department of Control of Neglected Tropical Diseases, World Health Organization, Avenue Appia 20, 1211 Geneva 27, Switzerland.; b Institut de Recherche pour le Développement, CIRAD, University of Montpellier, France.; c Institute of Tropical Medicine, Antwerp, Belgium.; d Animal Resources and Biosecurity, Makerere University, Kampala, Uganda.; e Foundation for Innovative New Diagnostics–Kenya, Nairobi, Kenya.; f Neglected Tropical Diseases Programme, Foundation for Innovative New Diagnostics, Geneva, Switzerland.; g Department of Parasitology, Institut National de Recherche Biomédicale, Kinshasa, Democratic Republic of the Congo.; h Communicable Disease Unit, World Health Organization Regional Office for Africa, Brazzaville, Congo.

## Abstract

Human African trypanosomiasis is a life-threatening parasitic infection endemic to sub-Saharan Africa. Around 95% of cases are due to *Trypanosoma brucei gambiense*, found in western and central Africa. Clinical signs and symptoms are nonspecific, current diagnostic tests are not sufficiently accurate, and parasitological confirmation of infection requires microscopic examination of body fluids and specialized techniques for concentrating parasites. Moreover, current treatment is not recommended on the basis of suspicion alone because it is not sufficiently safe. The availability of a simple and accurate diagnostic test to identify individuals harbouring parasites would widen treatment and help decrease disease prevalence. A subcommittee of the World Health Organization’s Neglected Tropical Diseases Diagnostics Technical Advisory Group has developed a target product profile for a diagnostic tool to identify *T. b. gambiense* infection. This tool should have a high sensitivity for detecting *T. b. gambiense* but be simple enough to use in rural Africa. Ideally, the tool could be applied by any minimally trained individual in an unsophisticated peripheral health facility, or a mobile team in a village with little infrastructure. The test should be able to function under hot and humid conditions. Basic training should take under 2 hours and the test should involve fewer than five steps. There should be no need for instrumentation or precision liquid handling. The test should yield a qualitative result in under 20 minutes that can be easily observed, and one test should be sufficient for determining treatment. A unit cost below 1 United States dollar (US$) would enable mass screening.

## Résumé

La trypanosomiase humaine africaine est une infection parasitaire potentiellement mortelle endémique en Afrique subsaharienne. Dans près de 95% des cas, elle est causée par *Trypanosoma brucei gambiense*, que l'on trouve en Afrique occidentale et centrale. Les symptômes et signes cliniques sont aspécifiques, les tests de diagnostic existants ne sont pas assez précis et la confirmation parasitologique de l'infection nécessite un examen microscopique des liquides corporels ainsi que des techniques spécialisées pour concentrer les parasites. En outre, il n'est pas recommandé d'entamer le traitement actuel sur la base d'une simple suspicion car celui-ci n'est pas suffisamment sûr. Fournir un test de diagnostic simple et précis permettant d'identifier les individus porteurs de parasites contribuerait à élargir le traitement et à une diminution de la prévalence de la maladie. Un sous-comité du Groupe consultatif technique sur les produits de diagnostic des maladies tropicales négligées de l'Organisation mondiale de la Santé a élaboré un profil de produit cible pour un outil visant à détecter une infection par *T. b. gambiense*. Cet outil doit être suffisamment sensible pour déceler la présence de *T. b. gambiense* mais suffisamment simple pour être utilisé dans les régions rurales du continent. Idéalement, il doit pouvoir être employé par toute personne ayant reçu une formation sommaire, tant dans des structures de santé périphériques basiques qu'au sein d'une équipe mobile dans un village doté d'infrastructures restreintes. Par ailleurs, il doit fonctionner dans une atmosphère chaude et humide. La formation requise pour son utilisation doit durer moins de deux heures et le test doit être effectué en moins de cinq étapes, sans exiger d'équipement spécifique ni de manipulation délicate. Cet outil doit fournir un résultat fiable en moins de 20 minutes, facile à interpréter, et un seul test doit suffire à établir un traitement. Enfin, afin d'organiser un dépistage de masse, son coût unitaire ne doit pas dépasser un dollar américain.

## Resumen

La tripanosomiasis humana africana es una infección parasitaria potencialmente mortal endémica del África Subsahariana. Alrededor del 95% de los casos se deben al *Trypanosoma brucei gambiense*, presente en África Occidental y Central. Los signos y síntomas clínicos no son específicos, las pruebas diagnósticas actuales no son suficientemente precisas y la confirmación parasitológica de la infección requiere el examen microscópico de los fluidos corporales y técnicas especializadas de concentración de parásitos. Además, el tratamiento actual no se recomienda a partir de la sola sospecha porque no es suficientemente seguro. La disponibilidad de una prueba diagnóstica sencilla y precisa para identificar a las personas con parásitos ampliaría el tratamiento y ayudaría a disminuir la prevalencia de la enfermedad. Un subcomité del Grupo de Asesoramiento Técnico sobre Diagnóstico de Enfermedades Tropicales Desatendidas de la Organización Mundial de la Salud ha desarrollado un perfil de producto objetivo para una herramienta de diagnóstico destinada a identificar la infección por *T. b. gambiense*. Esta herramienta debe tener una alta sensibilidad para detectar *T. b. gambiense*, pero ser lo suficientemente sencilla para su uso en las regiones rurales de África. Lo ideal sería que la herramienta pudiera ser aplicada por cualquier persona mínimamente capacitada en un centro sanitario periférico poco sofisticado o por un equipo móvil en un pueblo con poca infraestructura. La prueba debería funcionar en condiciones de calor y humedad. La formación básica debe durar menos de 2 horas y la prueba debe constar de menos de cinco pasos. No debe necesitarse instrumentación ni manipulación precisa de líquidos. La prueba debe dar un resultado cualitativo en menos de 20 minutos que pueda observarse fácilmente y debe bastar una prueba para determinar el tratamiento. Su coste unitario, inferior a un dólar estadounidense, permitiría un cribado masivo.

## ملخص

داء النوم الأفريقي البشري هو عدوى طفيلية مهددة للحياة متوطنة في جنوب الصحراء الكبرى بأفريقيا. يعود سبب حوالي %95 من الحالات إلى داء النوم البروسي الغامبي، الموجودة في غرب ووسط أفريقيا. العلامات والأعراض الإكلينيكية غير محددة، والاختبارات التشخيصية الحالية ليست دقيقة بشكل كاف، ويتطلب التأكيد الطفيلي للعدوى فحصًا مجهريًا لسوائل الجسم، وتقنيات متخصصة لتركيز الطفيليات. علاوة على ذلك، لا يُوصى بالعلاج الحالي على أساس الاشتباه وحده لأنه ليس آمنًا بشكل كاف. إن توفر اختبار تشخيصي بسيط ودقيق لتحديد الأفراد المصابين بالطفيليات، من شأنه أن يوسّع العلاج ويساعد في تقليل انتشار المرض. قامت لجنة فرعية من "المجموعة الاستشارية الفنية لتشخيص أمراض المناطق المدارية المهملة" التابعة لمنظمة الصحة العالمية، بتطوير ملف تعريف منتج مستهدف لأداة تشخيص لتحديد الإصابة بعدوى داء النوم البروسي الغامبي. يجب أن يكون لهذه الأداة حساسية عالية للكشف عن داء النوم البروسي الغامبي، ولكنها تكون بسيطة بما يكفي لاستخدامها في المناطق الريفية بأفريقيا. من الناحية المثالية، يمكن استخدام الأداة بواسطة أي فرد لديه الحد الأدنى من التدريب في مرفق صحي ثانوي غير متطور، أو فريق متنقل في قرية ذات بنية تحتية بسيطة. يجب أن يكون الاختبار قادرًا على العمل في الظروف المناخية الحارة والرطبة. يجب أن يستغرق التدريب الأساسي أقل من ساعتين، ويجب أن يشتمل الاختبار على أقل من خمس خطوات. يجب ألا تكون هناك حاجة لأدوات القياس، أو لمعالجة السوائل بدقة. يجب أن ينتج عن الاختبار نتيجة نوعية في أقل من 20 دقيقة، ويمكن ملاحظتها بسهولة، ويجب أن يكون اختبار واحد كافياً لتحديد العلاج. عندما تكون تكلفة الوحدة أقل من دولار واحد أمريكي، فهذا من شأنه أن يسمح بالفحص الشامل.

## 摘要

非洲人类锥虫病是撒哈拉以南非洲地区特有的一种危及生命的寄生虫感染。大约 95% 的病例是由在西非和中非发现的*布氏冈比亚锥虫*引起的。临床征兆和症状是非特异性症状，目前的诊断测试不够准确，确诊寄生虫感染需要对体液进行显微镜检查和专门的寄生虫浓缩技术。此外，不建议使用当前的治疗方法，因为它的安全性受到质疑。如果能有一种简单而准确的诊断试验来识别携带寄生虫的个体，这将扩大治疗范围，并有助于降低疾病患病率。世界卫生组织被忽视热带病诊断技术咨询组的一个小组委员会制定了用于诊断工具的目标产品简介，以识别*布氏冈比亚锥虫*感染。该工具应在检测*布氏冈比亚锥虫*病方面具有很高的灵敏度，但使用时也需足够简单，以便在非洲农村使用。理想情况下，该工具可由任何受过最低限度培训的个人在简陋的外围卫生设施中使用，也可由流动小组在缺乏基础设施的村庄中的使用。该测试应能在湿热条件下进行。基础培训应在 2 小时内完成，测试应少于 5 个步骤。应该不需要仪器或对液体进行精密处理。该测试应在 20 分钟内产生易于观察的定性结果，并且一次测试应足以确定治疗方案。如果单位成本低于 1 美元，就可以进行大规模筛查。

## Резюме

Африканский трипаносомоз человека представляет собой опасную для жизни паразитарную инфекцию, распространенную в Африке к югу от Сахары. Около 95% случаев заболевания вызваны *Trypanosoma brucei gambiense*, встречающейся в Западной и Центральной Африке. Клинические признаки и симптомы носят неспецифический характер, существующие диагностические тесты недостаточно точны, а паразитологическое доказательство инфекции требует проведения микроскопического исследования жидкостей организма и применения специализированных методов для концентрирования паразитов. Кроме того, в настоящее время не рекомендуется проводить лечение только на основании подозрений, поскольку его безопасность не является достаточно высокой. Наличие простого и точного диагностического теста для выявления носителей паразитов расширит возможности лечения и поможет снизить распространенность заболевания. Подкомитет технической консультативной группы Всемирной организации здравоохранения по диагностике забытых тропических болезней разработал целевой профиль продукта для диагностического инструмента с целью выявления инфекции *T. b. gambiense*. Этот инструмент должен обладать высокой чувствительностью для выявления *T. b. gambiense*, но быть достаточно простым для использования в сельских районах Африки. В идеале этот инструмент могли бы применять любой, минимально подготовленный специалист в необорудованном периферийном медицинском учреждении или выездная бригада в деревне с неразвитой инфраструктурой. Тест должен быть рассчитан на проведение в условиях жаркого и влажного климата. Начальная подготовка должна занимать менее 2 часов. Тест должен состоять не менее чем из пяти этапов. При этом необходимость в использовании измерительных приборов или точной обработке жидкостей должна отсутствовать. Тест должен давать качественный и понятный результат менее чем за 20 минут, и одного теста должно быть достаточно для назначения лечения. При стоимости единицы менее 1 доллара США становится возможным проведение массового скринингового обследования.

## Introduction

Human African trypanosomiasis is a life-threatening parasitic infection transmitted by the tsetse fly that is endemic in sub-Saharan Africa. Since the infection caused devastating epidemics during the 20th century, its incidence has fallen to historically low levels thanks to sustained and coordinated efforts over the past 20 years.^1^ Two trypanosome subspecies, with distinct epidemiological characteristics, cause the disease: (i) *Trypanosoma brucei rhodesiense*, which is found in eastern and southern Africa, is harboured by wild and domestic animals (its reservoir) and is transmitted occasionally to humans; and (ii) *T. b. gambiense*, which is found in western and central Africa, has its main reservoir in humans, and between 2011 and 2020 accounted for 95% (32 275 out of 34 096 reported cases) of the total caseload of human African trypanosomiasis.^1^

The diagnosis of human African trypanosomiasis relies on laboratory techniques because clinical signs and symptoms are nonspecific. Serodiagnostic tests for use in the field exist only for *T. b. gambiense*. However, as these tests are based on the detection of antibodies, they do not confirm the presence of infection. Given the current low disease prevalence, the positive predictive value of serological tests is particularly low. Diagnostic tools suitable for application in the field include: (i) the card agglutination test for trypanosomiasis, which is used mainly in active screening by specialized mobile teams; and (ii) rapid diagnostic tests, which are better suited to individual testing at the point of care. Parasitological confirmation of *T. b. gambiense* infection requires microscopic examination of body fluids by skilled personnel, which is labour intensive. The best-performing parasitological tests have a diagnostic sensitivity of 85–95% at best, but are more complex to perform than tests with lower sensitivity.^2^

For gambiense human African trypanosomiasis, it has been observed for many years that repeated rounds of serological screening followed by the treatment of any cases detected can bring the disease prevalence down to a low level. Consequently, this strategy has become the cornerstone of the control and elimination of gambiense human African trypanosomiasis. However, it is known that a variable proportion of seropositive but microscopically unconfirmed individuals harbours the parasite and could provide an ongoing reservoir for the disease. To date, treatment cannot be recommended on the basis of suspicion alone because current treatments are not sufficiently safe. The advent of a safer and easier-to-use treatment would favourably alter the benefit–risk balance, and would enable treatment to be widened to include individuals with a high degree of suspicion of harbouring parasites. The availability of a simple diagnostic tool that could identify individuals eligible for treatment would be the ideal complement. Together, a simple diagnostic tool and safe, easy-to-use treatment would provide a powerful means of eliminating the disease.

## Method

The World Health Organization’s (WHO) Department of Control of Neglected Tropical Diseases led the development of a target product profile for a diagnostic tool to identify individuals with a suspected, but microscopically unconfirmed, gambiense human African trypanosomiasis infection who are eligible for treatment with safe and easy-to-use medicines. During this process, WHO’s standard guidance for target product profile development was followed.^3^ A subgroup on the diagnostic needs of human African trypanosomiasis was established as part of the WHO Neglected Tropical Diseases Diagnostics Technical Advisory Group, which was formed to identify and prioritize the diagnostic needs of neglected tropical diseases. The Advisory Group of independent experts comprised leading international scientists and specialists, including from countries where the disease is endemic. Standard WHO declaration of interest procedures were followed.^4^ Initially, a landscape analysis of the products currently available and in development was conducted and salient areas of unmet need were identified. In a series of meetings and remote consultations, the subgroup identified several scenarios in which possible diagnostic tools could help fill the main gaps in disease treatment and control, and arranged these scenarios in order of priority. A template for the development of a target product profile for a human African trypanosomiasis test, which included adaptations to the disease context, was agreed. A draft of the target product profile (rated as priority no. 2) underwent several rounds of review by subgroup members between March and November 2021. The ensuing version was reviewed by Neglected Tropical Diseases Diagnostics Technical Advisory Group members, and draft version 0.1 was posted on WHO’s website for 28 days in November and December 2021 for public consultation with a comment form.

This new diagnostic tool should have a high sensitivity for detecting *T. b. gambiense* infection but be simple enough to be applicable at point-of-care settings in rural Africa. The disadvantages of the sensitive parasitological methods currently used to confirm infection are that they require specialized materials and techniques for concentrating parasites, which often depend on having a source of electricity, and which may be unavailable at the point of care in regions where human African trypanosomiasis is endemic. It is possible that easier-to-use parasitological methods could appear in the future. The envisioned tool could be in any format, so long as it is simple and involves little specialized training.

In addition, any new diagnostic tool should be able to identify individuals, irrespective of symptoms, in whom the likelihood of infection is sufficiently high to justify treatment with a medicine with a good safety profile. Ideally, it should be possible to reach a therapeutic decision after one test, though a tandem of two simple sequential tests would also be acceptable. In addition, the test should be usable in unsophisticated peripheral health facilities located in or near communities at risk of human African trypanosomiasis, and by mobile teams that visit villages with little infrastructure.

A test satisfying these requirements would be valuable even in the absence of a safe and easy-to-use anti-trypanosome treatment, which is expected to be developed. At present, the safety and mode of administration of the medicines available are not appropriate for extended use in individuals who are suspected of having human African trypanosomiasis, but in whom the presence of parasites has not been confirmed. When a safe, effective and simpler anti-trypanosome treatment does become available, it would be conceivable to widen the eligibility criteria for treatment to include individuals without parasitological confirmation but with a high degree of suspicion of harbouring parasites. Such widened treatment would benefit infected individuals in whom current diagnostic methods are unable to confirm an infection. Simultaneously, the community would benefit as some human parasite reservoirs that perpetuate the risk of transmission would be further suppressed.

## Target product profile^5^

### Intended use

A diagnostic tool for detecting *T. b. gambiense* infection should at a minimum be capable of detecting infection by any species of the subgenus *Trypanozoon* or, preferably, by *T. b. gambiense* itself. A qualitative test that can detect *T. b. gambiense* antibodies, antigens or nucleic acids or the whole parasite would be ideal, but one that detected *Trypanozoon* antibodies, antigens, nucleic acids or whole parasites would be sufficient. Today, if any type of trypanosome is observed microscopically in human body fluids in a region where gambiense human African trypanosomiasis is endemic, the infected individual will receive treatment even though the subspecies may be unknown. It has to be borne in mind that antibodies can linger from a previous infection. Whole parasite detection would overcome this problem, and lens-free optical detection methods are currently being investigated. 

The diagnostic test is intended for use in individuals at risk of gambiense human African trypanosomiasis. Specimens should be collected without discomfort to the individual that is disproportionate to the health benefit and, preferably, non-invasively. Possible non-invasive tests may sample, for example, saliva, urine or tears, and possible minimally invasive methods include the finger prick test and skin microbiopsy. Techniques that do not involve specimen collection may be possible.

Ideally, the test should clearly identify individuals with suspected gambiense human African trypanosomiasis suitable for treatment but, at a minimum, it should be able to preselect individuals. A single test would be ideal but a second sequential test, at most, may be needed to narrow down the selection. A positive test result should trigger treatment. In addition, specimens should be collected and sent for remote assessment using a test with higher specificity to monitor the epidemiological status of human African trypanosomiasis in the region.

In practice, the test should be capable of being applied by any minimally trained individual working either at a peripheral health facility or in a mobile team in a village with little infrastructure. At a minimum, it should be capable of being applied by a minimally trained laboratory technician working at a first-line peripheral health facility that refers patients testing positive to a higher-level laboratory for further assessment. The closer testing is performed to the communities at risk, the better.

### Test performance

The test should be at least as sensitive as most parasitological tests currently in use because individuals with a false-negative result will not be treated, with possible fatal consequences, and the disease incidence will be underestimated. At a minimum, the test should have a sensitivity and specificity above 95% for detecting members of the subgenus *Trypanozoon*. Ideally, it should have a sensitivity and specificity above 99% for detecting *T. b. gambiense* type 1 and type 2. The specificity required will depend mainly on the safety of the medicines available: the less safe, the higher the specificity needed. False-positive results can lead to unnecessary treatment and to overestimates of disease incidence. In addition, the analytical sensitivity of the test should ideally be equivalent to 10 or fewer parasites per mL of blood or, at most, 100 parasites per mL. Tests that can detect antigens or nucleic acid sequences may achieve lower detection thresholds than those detecting whole parasites. At present, in endemic regions, diagnosis and treatment are based on microscopic identification of protozoa of the subgenus *Trypanozoon*.

The repeatability of the results of different tests performed on the same sample by the same reader using the same instruments in the same environment should have a *Κ*-value above 0.92 (ideally above 0.96). Similarly, the reproducibility of the results of different tests performed on the same sample by the same or a different reader using a different instrument or in a different environment should have a *Κ*-value above 0.90 (ideally, above 0.94). 

Quality control will depend on the test format. At a minimum, each test should include a control of functionality to indicate it is performing properly. Ideally, there should be a control of individual functionality for each test and positive and negative controls for batch testing and possibly for kit testing. Positive and negative controls must be temperature stable. It would also be useful to have a proficiency panel, which is a collection of specimens of known reactivity that is used to check diagnostic tests against a standard.

### Regulatory requirements

At a minimum, the test’s components should be manufactured in accordance with Current Good Manufacturing Practice (GMP) or ISO 13485:2016. Preferably, manufacturing should be in accordance with CE marking, and compliant with the European Union’s Directive 98/79/EC (IVDD 98/79/EC) and ISO 13485:2016 for the quality management system (QMS). However, the new CE marking rules are more demanding and may entail unrealistic production costs. In any case, the quality management system should be clearly defined. In addition, the test must be commercially available on the market. There are no particular requirements for promotional or marketing material.

### Health-care system needs

#### Test operation

Ideally, the test should be able to be used at a temperature between 10°C and 40 °C and a relative humidity between 10% and 88%. The minimum requirement is an operating temperature range of 10°C to 30 °C and a relative humidity range of 40% to 70%. Preferably, the test should involve fewer than five steps and there should be no need for precision liquid handling. At a minimum, there should be fewer than 10 steps and the use of simple pipette devices only. Ideally, the result should be available in under 20 minutes or, at a maximum, under 2 hours.

#### Instrumentation

Ideally, the test should not require instrumentation. However, if instrumentation is required, it should: (i) be portable or hand-held; (ii) weigh 5 kg or less; (iii) be durable, such that it can be easily and safely transported to the field; (iv) be battery-operated and able to run on standard mains electricity; (v be able to function without the need for running water; (vi) be resistant to shock and vibration; (vii) be easy to maintain; and (viii) have a life span of at least 5 years. The total cost of any instrumentation or devices required to perform testing should take into account the need to perform tests in first-line facilities, even with a possible lack of infrastructure.

#### Data recording and transmission

The test should yield a qualitative result that can be observed visually or is displayed on a portable device. Ideally, the result should be stable for at least 30 minutes. At a minimum, it should be stable for at least 15 minutes. No digital interface or electrical connectivity should be required to obtain the result.

The result could be recorded in a logbook or on a computer or smartphone. Ideally, the result should be capable of being integrated into national data and reporting systems and of being easily stored for retrospective interpretation (e.g. electronic results, optical density or intensity measurements, or electronic images or video). However, treatment decisions should not depend on having data connectivity. The data recorded should include test results and demographic information and should be exportable to any database, if necessary. The amount of data storage needed will depend on the program used.

Ideally, the data generated should be capable of being automatically integrated into a database on a server without the need for additional equipment. At a minimum, the test results could be entered manually into a computer database and transmitted manually. Data transmission should be flexible, such that, depending on connectivity, data could be sent by email, Short Message Service (SMS) or phone. The data format should be compatible with existing health-care databases, such as JavaScript Object Notation (JSON, Ecma International, Geneva, Switzerland) or District Health Information Software 2 (DHIS2, University of Oslo, Oslo, Norway), and should facilitate seamless transmission to these databases if required.

#### Test stability and handling

All tests should be packaged individually and accompanied by any accessories required for sample collection and processing as well as by operating instructions and bench aids. Ideally, the test should be stable at a temperature between 4 °C and 45 ᵒC and a relative humidity between 40% and 88% for at least 24 months, and be able to withstand transportation at 50 °C. Preferably, transportation should not require a cold chain. At a minimum, the tests should be stable at a temperature between 4 °C and 8 ᵒC and a relative humidity between 40% and 88% for at least 12 months. Stability estimates should take into account the time needed for transport from the manufacturer, passage through customs and local distribution. In use, the test should ideally be stable for at least 2 hours after the test pouch is opened or, at a minimum, for 30 minutes after opening. Ideally, reagents should be ready to use or require a maximum of two additional steps to be ready to use; at a minimum, they should be ready to use within 15 minutes and should require no more than five additional steps.

#### Sample handling

The volume of the specimen will depend on the type of sample. Ideally, blood would be collected by finger prick or capillary tube, with a maximum volume of 0.07 mL. Alternatively, blood, serum or plasma collection would require a maximum volume of 5 mL. Additional specimens could be collected at the same time for repeat or remote testing if needed. The collection of samples of any other body fluids or tissues should require minimal effort.

Ideally, collecting devices should be provided with the test kit and there should be minimal or no specimen processing. Alternatively, routine collecting devices could be used, with minimal specimen processing. However, it is important to note that special collecting devices are not available routinely in peripheral health centres in regions where human African trypanosomiasis is endemic. Occasionally, left-over specimens could be preserved and transported under certain conditions.

Standard biosafety precautions for handling potentially infectious materials should be taken. Waste, including sharp objects and containers for lancets and capillary tubes, should be disposed of in a biosafety bin according to standard guidelines. Excess specimens and consumables used in the test process should be disposed of using an appropriate method, for example, in a latrine or by incineration. Standard operating procedures should be provided.

#### Training and support

Ideally, basic training to use the test should take less than 2 hours or, at a maximum, 1 day. Manufacturers should replace non-functioning tests or instruments, and guarantee the supply of tests for at least 7 years after marketing (5 years at a minimum). External support should be available, with a response time of 1 day (1 week at a maximum).

### Sustainability and cost

Tests should continue to be produced even in the absence of a profitable market. As the testing and treatment of human African trypanosomiasis is a non-profit endeavour, funding has to be sustainable and an innovative model for test production and access is needed. Donors could ensure affordability. However, advocacy will be needed.

The cost of each test, excluding specimen collection costs, should ideally be 1 United States dollar (US$) or US$ 20 at a maximum. This amount does not include the cost of hardware, material shipment, sample collection or salaries. A US$ 1 test could be used for mass screening, whereas a US$ 20 test would instead be a second-line test.

## Conclusions

The human African trypanosomiasis subcommittee of WHO’s Neglected Tropical Diseases Diagnostics Technical Advisory Group has developed a target product profile for a diagnostic tool to identify the presence of *T. b. gambiense*. The availability of an affordable, easy-to-use, sensitive and accurate test would benefit individuals, who would receive curative treatment for this life-threatening disease. In addition, the whole community would benefit because treatment could be given to individuals whose infection cannot be confirmed by current diagnostic methods. The resulting wider treatment would eliminate parasite reservoirs, which would decrease transmission of the disease via the tsetse fly vector.
